# FAIR foundations of a novel indicator vault for non-communicable diseases in the European Union: feasibility study for effective contextualisation of indicators

**DOI:** 10.3389/fdgth.2025.1685733

**Published:** 2025-12-16

**Authors:** Iztok Štotl, Fabrizio Carinci, Stephen Fava, Astrid Lavens, Jana Lepiksone, Massimo Massi Benedetti, Tamara Poljičanin, Scott Cunningham, János Sándor, Nicholas Nicholson

**Affiliations:** 1 Department of Endocrinology, Diabetes and Metabolic Diseases, University Medical Centre Ljubljana, Ljubljana, Slovenia; 2 Faculty of Medicine, University of Ljubljana, Ljubljana, Slovenia; 3 Unicamillus International Medical University, Rome, Italy; 4 Department of Medicine, Mater Dei Hospital of Malta, Msida, Malta; 5 Department of Medicine, University of Malta, Msida, Malta; 6 Health Services Research, Sciensano, Brussels, Belgium; 7 Department of Research and Health Statistics, The Centre for Disease Prevention and Control, Riga, Latvia; 8 Hub for International Health Research, Perugia, Italy; 9 Zagreb County Health Center, Samobor, Croatia; 10 Clinical Technology Centre, Level 7, Ninewells Hospital, Dundee, United Kingdom; 11 Department of Public Health and Epidemiology, School of Health Sciences, University of Debrecen, Debrecen, Hungary; 12 European Commission, Joint Research Centre (JRC), Ispra, Italy

**Keywords:** health indicator framework, non-communicable diseases, indicator contextualisation, FAIR, SOLICIT, EHDS, health indicator, metadata

## Abstract

**Background:**

Comparing health indicators across the European Union (EU) is a challenging endeavour. A feasibility study was conducted to explore opportunities for improvement through the contextualisation of indicators for major non-communicable diseases (NCDs). We aimed to improve the usability and transparency of indicators in the domain of NCDs by describing the contextual information about the data from which they draw and the related data processes. In particular, we sought to illustrate how semantic linkage could be achieved to facilitate interoperability with other metadata models using FAIR data principles. Finally, we aimed to provide recommendations for the implementation of the proposed metadata model at the EU level.

**Methods:**

A number of expert group meetings were held between March 2023 and October 2024 to agree on the approach and related technologies to meet the standard requirements for the meaningful comparison of indicators across countries and regions of Europe in the domain of NCDs.

**Results:**

The Semantic Ontology-Labelled Indicator Contextualisation Integrative Taxonomy (SOLICIT) was selected as a suitable generic metadata model for contextualising indicators. In this work, we adapted the SOLICIT generic framework to the diabetes sub-domain and extended its applicability more generally across all NCDs. As a proof of concept, we present an example of how to adapt a diabetes indicator and its related contextualisation within SOLICIT.

**Conclusion:**

The accurate contextualisation of NCD indicators can substantially improve their use and comparability across national and regional boundaries. This study delivered a set of seven recommendations for implementation in three different areas: (a) contextualisation of common data elements and indicators (use of contextual information; common schema for semantic linkage), (b) generic contextualisation framework (adoption of the framework; use of SOLICIT), and (c) implementation at EU level (pilot test of the model on federated **networks**; development of European portals; implementation of a user-friendly interface for SOLICIT). The proposed concepts provide a way of validating indicator values and their comparisons, as well as their provision, including all relevant details, encouraging secondary use and potential integration with additional indicator sets. Further studies are needed to test and refine the proposed model.

## 1 Introduction

Harmonising indicators is important for comparison and benchmarking purposes, but the process is far from straightforward and particularly challenging in the heterogeneous backdrop of European Union (EU) health service infrastructures (1). The derivation of an indicator generally requires several data processing stages, and yet the provenance of the data as they propagate through these stages is rarely available. As a consequence, it is difficult to validate indicators and/or to reuse them outside their specific purpose without this information (2).

The classic definition of metadata is based on the etymology of the word itself—metadata is “data about data” (3). Most definitions from past literature describe metadata as a formal representation of data that describes them in a (preferably) standardised, stable, and unambiguous way (4). The use of metadata is an essential element for the integration of heterogeneous data sources to achieve a valid and meaningful data fusion, enabling a comprehensive reuse of the associated information (5).

In the context of non-communicable diseases (NCDs), the definition of “common data elements” (CDEs) represents a key step in the formulation of harmonised metadata components. The National Institutes of Health define a CDE as “a data element that is common to multiple data sets across different studies” (6) and contributes to making data interoperable (7). They can also be seen as the fundamental elements that must be processed according to specific technical specifications to deliver an indicator.

CDEs and indicators both require robust metadata. For instance, recording systolic blood pressure as a value does not provide sufficient insight into the circumstances in which the measurement occurs. Using metadata, information can be included concerning the state of the subject during the single measurement, as well as the protocol and circumstances under which the measurement was made, together with the recommended personal target level or associated guideline (8). Other information might be added, such as pulse pressure and details concerning how the measurement was taken—for example, by a healthcare facility or by the person themselves (as in a survey). Whereas the recording of all this information is not mandatory, it can add an extra degree of usefulness when available and help ascertain sources of bias.

Indicators can be contextualised similarly using metadata. For instance, many parameters can be added to an indicator measuring the rate of diabetes in a given country, such as the definition and purpose of the indicator, the subjects of the indicator, indicator quality, and data provenance. Metadata can also describe the process used for integrating heterogeneous data sources used for the calculation of an individual indicator value. Such enriched indicator information provides significantly greater value to the observer than a mere numeric value.

Composite indicators pose particular challenges since they incorporate a complex set of relations into single values, presenting a series of theoretical and methodological challenges. Assumptions need to be assessed carefully to avoid producing results of dubious analytic rigour (9) and “slippery indicators” that do not measure what they actually claim (10). This requires care throughout the whole process chain, from data storage and integration, to their processing and aggregation to produce meaningful and comparable indicators (9).

Indicators are generally derived using an agreed protocol (e.g. a standard algorithm or derivation method) on a set of CDEs relevant to the specific target. A relevant example that involves longitudinal statistical analysis is the incidence rate of a given disease in a specific geographical area over a defined time period. There may be many critical factors to consider, such as whether all the related data elements are complete and available from a single or multiple data sources. In the case of heterogeneous data sources, there is generally a need to apply more advanced algorithms to control for bias and enhance the comparability of results. Moreover, the selected derivation method may not be applicable as specified under certain conditions.

All the above choices contribute to the specification of a “context” for each indicator, which is important to know and record—especially if the indicator might eventually be compared with similar others across regions or countries. Such contextual information can be used to support additional comparisons of quality and reliability for the same indicator.

The “good indicators guide” (11) of the British National Health Service Institute for Innovation and Improvement emphasises that a poorly designed or poorly chosen indicator with reliable data, or a well-designed indicator with unreliable and/or untimely data, has very little value and can even be dangerous. The guide also stresses the importance of metadata to help understand if the indicator is relevant for a particular purpose and whether it is populated with reliable data. These criteria underscore the importance of adding a contextualisation layer to each indicator within a framework that allows comparing and linking these results under different circumstances.

This method of working is required by the recent regulation underpinning the creation of the European Health Data Space (EHDS) (12). According to its specifications, the EHDS will provide a data discovery function via a federated system of data catalogues providing metadata descriptions of individual data sets. It will also provide a seamless mechanism allowing data users to request and receive data (dependent on the necessary data processing agreements) from data sources independent of where they are located.

The present paper is based on the experience of an international forum promoted by the European Commission to undertake firm steps in this direction. The “collaborative health information European framework” (CHIEF) is an initiative of the European Commission coordinated by the Joint Research Centre in collaboration with the Directorate-General for Health and Food Safety (DG SANTE), to address new ideas and solutions to overcome the difficulties and hindrances towards the regular collection of NCD indicators in Europe (13). Metadata represents one of the three main points of focus that have been tackled to improve the use of available data through more accurate data descriptions.

In the EU, previous projects have shown the viability of a cohesive infrastructure, highlighting challenges at both the organisational and technical levels (14).

Technical challenges include the harmonised specification and contextualisation of indicators and harmonised statistical tools, particularly in a federated network, for deriving indicators from different types of data sources. Organisational challenges include the processes needed for agreeing on the sets of indicators viable in the setting of a given NCD domain and for defining the domain-related elements for the derivation of the indicators within the domain. Given the cross-disciplinary nature of indicators, the aspect of a collaborative network of specialists in a given disease domain is critical for defining and agreeing on the indicators most relevant for the domain (15). The limitations of the data collection and data processing methods arising from a wide range of heterogeneous processes and the subsequent contextualisation of indicators derived from them can only be fully understood within the collective work of a representative group of experts cognizant of all the underlying issues and effects.

CHIEF seeks to address the challenges of the above dimensions, identifying specific directions through which the implementation of the EHDS could be accelerated. One of the specific interests of experts involved in CHIEF has been how to provide efficient data that can be integrated with the collaborative work already organised within registry networks (RNs).

These data may be provided in the form of harmonised data records (where data privacy agreements allow) or as aggregated harmonised data, epidemiological indicators, and epidemiological analyses. These data sets can be listed within the EHDS data catalogue and made accessible via the EHDS data retrieval mechanisms. The framework addressed by CHIEF can complement and strengthen the mechanisms foreseen by EHDS by standardising the way in which processing instructions can be provided by data users to the individual data providers.

In this way, a complex process (15) may be more effectively realised within the standardised ecosystem of RNs, which is structurally embedded into CHIEF, thus already able to reinforce the contents of the EHDS, which is largely agnostic of such considerations.

The structuring and contextualisation of data are critical for ensuring appropriate use of the underlying data for epidemiological purposes, where the interpretation of results derived from heterogeneous data sources is only meaningful with a sufficiently comprehensive overview of the data sources themselves and their limitations. This is particularly the case for secondary data use, where data users may not necessarily be experts within a given disease domain.

To fully enable open data opportunities, digital data assets within a general framework, e.g. the one advocated by CHIEF for NCDs, must comply with FAIR principles, ensuring findability, accessibility, interoperability, and reusability (2). These principles prioritise machine-actionability, as modern data challenges (volume, complexity, and velocity) make computational support essential for human users. The technical framework for structuring, contextualisation, and validation of the data within the RNs would serve to add considerable value to CHIEF's data and health indicators and help motivate a business model for sustainability.

The general objective of the present study is to support the activity of CHIEF through the identification of a suitable generic metadata model that could be applied across NCDs for describing the indicators and the data from which they draw.

The specific objectives are the following:

- to demonstrate the applicability of the model in terms of capacity to handle structured metadata, with an emphasis on reuse, retrieval of contextual information at any degree of granularity, and propensity to incorporate different quality metrics;
- to describe the conceptual model using semantic linkage for all components;
- to demonstrate its interoperability with other metadata models, using FAIR data principles to describe how automatically mapping between similar variables may be covered by different terminologies, and providing a practical example of how a diabetes indicator would be described; and
- to provide final recommendations to the EU for the implementation of the metadata model.

## 2 Materials and methods

We carried out an expert revision coordinated by the European Commission's Joint Research Centre between 2022 and 2024. The specification of the metadata model involved the formation of a subgroup of the CHIEF “diabetes design working group” (CHIEF-diabetes.dwg), an expert group formed by the European Commission to discuss, agree, define, and test a basic set of harmonised indicators at the EU level. The other points of focus addressed by the group included: selection of indicators, federated analytics, privacy and ethics, and stakeholder engagement.

To ensure practical applicability and real-world relevance, the CHIEF consultations were conducted by a multidisciplinary team of experts, including medical doctors, public health experts, biostatisticians, and ontology and eHealth specialists (comprising all authors of this paper). This approach ensured that sufficiently diverse views and cross-functional expertise could be deeply embedded in the solutions designed from the outset, refining the methodology according to the needs, to strengthen the content of the final recommendations.

An expert review was undertaken via a number of meetings held between March 2023 and October 2024. Five group calls were conducted during the study period. The work was subdivided into different clusters: ontology framework, registry network, and regional node description; data sources metadata; indicator metadata; and quality metadata framework. A number of intermediate work documents for fostering debate were produced during the work period.

Regarding the contextualisation framework, it was agreed that a suitable indicator contextualisation framework requires the basis of some standard and the ability to define metadata at different domain levels that could be defined and reused in a scalable way. A framework that could be implemented at a federated level was also considered important, as well as the ability to link metadata terms to standard terminology servers. These group meetings were supplemented with extensive reviews of existing literature to consider possible solutions.

The appropriateness of the proposed framework was evaluated against its ability to provide cover for the essential metadata elements of an indicator as described in the “good indicators guide” (11).

### 2.1 Applied standards, methodologies, and resources

The first step in the group meetings was to consider the availability of appropriate methodologies and standards and thereafter to agree on the ones to use and adapt for the indicator contextualisation needs. To provide the required degree of flexibility and scalability, a metadata model for contextualising indicators has to draw on several concepts, methodologies, standards, and resources. A brief description of some of the main elements is provided below.

Metadata describes and constrains specific data objects (table fields, attributes of form questions, records, etc.) with descriptive elements, such as data type, range, and value ranges. Single units can be integrated into more complex elements that are often called data elements following the metadata registry standard ISO/IEC 11179 (16) of the International Organisation for Standardisation (ISO). ISO/IEC 11179 makes a distinction between the concept of a metadata element (i.e. the data element concept) and its actual representation in terms of its value (i.e. the value domain, which specifies the specific unit of measurement; the data type such as string, integer, and date; and the allowed range of values). This distinction helps separate the metadata describing the data element from the data values.

Metadata can be stored centrally in metadata repositories or data dictionaries (both terms are used synonymously) (17) and can have bindings to terminologies, controlled vocabularies, and taxonomies. The use of metadata enables the separation of content information from layout information (18) and can facilitate data processing operations without human guidance based on detailed machine-readable and actionable descriptions (19). With the advancement of information technologies, the acquisition of metadata has gradually become a critical step in building independent and knowledge-based information systems. The term “metadata methodological commons” refers to a series of metadata-related methods with the automatic processing of semantic formalisation as the next important step for the adoption of metadata methodological commons for the future needs of the artificial intelligence era (20).

In practice, metadata collections are often poorly standardised (21), which compromises their usefulness for data intercomparison purposes, and adherence to a metadata standard is important for achieving data interoperability. Data interoperability can be further enhanced by the use of ontologies, which complement standardised metadata via the incorporation of semantic relationships and axioms of description logic.

In particular, ontologies provide formal descriptions of knowledge using sets of concepts and semantic relationships that hold between them (22). Concepts comprise such things as classes and instances of classes (or individuals); relationships describe attributes and semantic links. Rules can be created using axioms referring to these concepts and relationships. Moreover, the rules can be described formally by description logics (DLs) that form a foundational basis of ontologies. DLs are a reduced set of first-order logics that can be used to make automatic inferences on the ontology axioms. As a result, ontologies not only introduce a shareable and reusable knowledge representation but can also provide new, inferred knowledge about a given domain. Furthermore, the relationships between concepts are not necessarily restricted to a single ontology but can be used to link knowledge across ontologies.

Whereas ontologies and metadata standards have their individual strengths, they are fully complementary, and it is in combination that they provide the most versatility. Ontologies define semantic relationships, while metadata standards ensure uniformity of metadata representation—without reference to a metadata standard, an ontology would lead to interoperability issues between domains, especially if each domain created its own metadata schema, and without ontologies, a metadata schema would have to implement its own classification hierarchy that would not easily scale across domains and would likely introduce ambiguities in metadata terms used in different contexts within different domains (cf. the term “actor” in an IT-related domain and in a theatrical arts domain).

An international standard dedicated to the description of data element metadata is the ISO/IEC 11179 standard for metadata registries (23). The aim behind ISO/IEC 11179 is to provide a “general description framework for data of any kind, in any organisation, and for any purpose” (24), as well as to ensure users and owners of data have a common understanding of the meaning and descriptive characteristics of those data. The concepts of the ISO/IEC 11179 metadata model automatically serve to make data adhere to FAIR data principles (2), especially those addressing findability, interoperability, and reusability.

### 2.2 Selection of a generic framework

A promising data annotation framework based on an integration of the concepts of the ISO/IEC 11179 metadata registry standard and the mid-level ontology suite of the common core ontologies (CCO) (25) has been created recently: Semantic Ontology-Labelled Indicator Contextualisation Integrative Taxonomy (SOLICIT) (26). SOLICIT essentially allows any degree of contextualisation to be attributed at the level of CDEs. It was developed primarily for indicator contextualisation but is agnostic of any specific indicator domain. SOLICIT is a generic data contextualisation framework intended to be implemented as a hierarchy of ontologies mapping to the specific domain hierarchies.

Whereas other ontologies have been developed to facilitate semantic interoperability between component descriptions, they have all generally proceeded with specific goals in mind. Included in these are the I-ADOPT Framework Ontology (27), Function–Behaviour–Structure (FBS) Ontology (28), and Descriptive Ontology for Linguistic and Cognitive Engineering (DOLCE) (29). I-ADOPT was developed primarily in view of accurately encoding what is measured, observed, derived, or computed in relation to the Earth systems. Since it does not derive from a foundational ontology, it may eventually introduce mapping overheads between common, core concepts. FBS is essentially driven by its view of the world in the three ontological categories of function, behaviour, and structure and does not fit very well with the needs of a more general description framework. DOLCE provides a foundational ontology that is more complex than BFO but currently misses a mid-layer ontology such as CCO provides. Moreover, BFO is used extensively in biomedical applications and is a core element in the Open Biological and Biomedical Ontology (OBO) Foundry. Other examples are domain-specific ontologies that are tailored to non-clinical domains like urban systems (30, 31). Even meta-modelling approaches (32), which support general indicator reasoning and key process indicators, lack the specific semantics required for NCD phenotypes and intervention pathways.

SOLICIT integrates the composite metadata concept of ISO/IEC 11179 with the semantic relationship functionality afforded by ontologies (using CCO as the guiding template) to provide the contextualisation concept. SOLICIT extends the ISO/IEC definition of a data element to an indicator and incorporates further attributes using the CCO semantic relations. Thus, a SOLICIT-derived indicator consists of an ISO/IEC 11179 object class, property, and value domain as well as a set of standard attributes, some of which are of an archetype nature that can be replicated to build up as comprehensive a set of contextualisation fields as necessary following the philosophy of a dual-model type methodology (cf. Section 4.1). The latter is an important consideration to avoid frequent changes to the SOLICIT domain ontologies since any required extension of contextual information depends only on stable, generic concepts.

ISO/IEC 11179 also provides a mechanism whereby metadata items can be classified according to some external classification scheme, allowing the means to link to data dictionaries, thesauri, ontologies, and other terminological systems (e.g. SNOMED, LOINC, and OHDSI). Such a classification mechanism not only helps reduce duplication of terms but also promotes alignment and mapping of metadata for improved interoperability.

Added strengths of SOLICIT are that it is able to handle data quality metrics and incorporate many other ontologies into its schema (33).

## 3 Results

Considering the general lack of availability of suitable frameworks to cater to the complex needs described in the introduction, and especially ones integrating the various concepts described in Section 2.1, the group agreed to use the SOLICIT framework (26) as a suitable generic metadata model for the domain of NCDs for describing the indicators and the data from which they draw.

The collaborative work led to the definition of study cases, leading to the practical use of SOLICIT within CHIEF. In the context of NCDs, its use requires extending the SOLICIT generic framework first to the NCD general domain level and thereafter to the specific NCD sub-domain of diabetes. The advantage of this approach is that all the NCD commonalities would be shared by all NCD sub-domains, greatly promoting reuse and harmonisation of metadata. We provide an example of a diabetes indicator containing contextualisation information described in terms of SOLICIT to show how an indicator represented in this way can address the essential information of the indicators and meet the requirements laid out in the good indicators guide.

To show how a diabetes indicator can be contextualised using the SOLICIT framework, an example is taken of an indicator measuring the number of lower limb amputations for persons with diabetes in a given population. This indicator is a proxy measure for the efficacy of vascular care. A relevant discussion on the validity of this indicator is found in the paper of Kolossváry et al. (34).

For such an indicator to be informative to data users who may not be aware of the underlying processes involved in its construction, a number of contextual elements should be minimally available and readily accessible.

The expert group selected the following elements of the “good indicators guide” for inclusion:

1. Definition of the indicator (ideally described in formal terms to avoid the ambiguity of natural language)
2. Explicit rule(s) or algorithm(s) involved in its derivation
3. References to the specific underlying data sources
4. Relevant measures, e.g. numerator and denominator for a ratio or percentages
5. Measurement unit
6. Geographical area and relevant time period
7. Specific endpoint for which it is a proxy

Given that the SOLICIT framework is encapsulated in an OWL ontology, the SOLICIT components can be described semantically in terms of Resource Description Framework (RDF) triples. RDF triples are composed of three entities described by subject, object, and predicate (verb). This allows an interrelated network of entities to be constructed (an object in one relationship may become a subject in another relationship).

The ways in which SOLICIT can address the 10 questions recommended by the good indicators guide are described in Table 1 (11).

**Table 1:** Summary of how the SOLICIT framework can address the ten basic questions to be satisfied by an indicator adhering to the good indicators guide.

#	Question to address	The specific means by which SOLICIT provides the information
1	What is being measured?	Information provided by the data element concept (object class and property) of the indicator itself and by the variables involved in the stratification of the indicator. In the example of Figure 1, the data element concept is the conjunction of Crude Rate with the Diagnostic Property, and the two stratification variables are geospatial code and year; further information is available from the instance of the contextual data element class dealing with indicator scope (Figure 3)
2	Why is it being measured?	Information provided by an instance of the *indicator scope* data element class that could also include qualification aspects such as stakeholders, policy/social context, and research question
3	How is the indicator actually defined?	The definition is another context metadata element (cf. Figure 3). It may also be understood from the full complementarity of the indicator's semantic relations. It may be made as comprehensive as needed and extracted to populate a standard template. The definition may even be formalised using description logic, forming part of the OWL class axioms
4	Who are the subjects of the measurement?	Information provided by the attribute has numerator (Figure 2). Further qualified by the attribute occupies spatiotemporal region (Figure 3) (providing the overall geospatial code value), together with the indicator stratification variables
5	What time period does the indicator cover?	Information provided by the attribute *occupies temporal region* (providing the overall temporal code value, Figure 3), together with the indicator stratification variables (e.g. in the example of Figure 1, the stratification variable corresponding to year values)
6	What is the unit/dimension of the measurement?	Information provided by the value domain of the indicator itself, with the unit/dimension of the individual stratification variables provided by their own specific value domains
7	Where do the underlying data come from?	Information provided in a general way in the instance of the *data element* class dealing with indicator scope. More granular information can be provided using the dereferencing process described under question #8
8	How accurate and complete are the data?	Information provided by the derivation and derivation rule classes and the associated bias class. The derivation class references the data elements from which the indicator is derived (cf. the lower half of Figure 1). Each constituent data element also contains contextual information on derivation and bias, allowing a recursive reconstruction of individual biases of which the indicator is comprised. In addition, each data element can be traced back to the underlying data source that itself can be described in terms of a set of data quality metrics
9	Are there any caveats/warnings/problems?	Information provided by any number of contextual data element classes (cf. Figure 3). Data and indicator bias provided by instances of the *Bias* class referenced by the relation *realizes* (cf. Figure 1)
10	Are particular statistical tests needed based on the datasets used?	This question is arguably better addressed by the data user on the basis of the intended use of the data. The contextual information provided in a framework like SOLICIT can help the data user understand what statistical tests may be necessary for the intended use

In this way, CHIEF may allow that one indicator, say “i1” (subject) “is derived from” (predicate) a data source ds1 (object), and that the data source ds1 (subject) “was created from” (predicate) a survey s1 (object), and that the survey s1 (subject) “covered a” (predicate) time period t1 (object), etc. In this way, it is possible to traverse the whole RDF graph in a “follow-your-nose” sense to build up a comprehensive picture of the network. There is, in effect, no restriction on the amount of information that can be added to an RDF graph. The information in an RDF graph is captured in an RDF triplestore (essentially a database of RDF triples).

OWL ontologies use this principle to encapsulate the knowledge of interest in a given domain. OWL ontologies consist of a set of axioms (comprising classes and relations), and these may be directly stored in an RDF triplestore. Whereas ontology developers are free to invent their own names for all the entities in their ontologies, doing so will soon lead to interoperability issues, especially for entities common across ontologies. It is therefore advisable to import common entities and relations from foundational ontologies. SOLICIT follows this philosophy by using the base-layer ontologies and the mid-layer ontology suite. It is also careful to limit the amount of overly specific relational terms and relies on generic relations instead.

**Figure 1:**
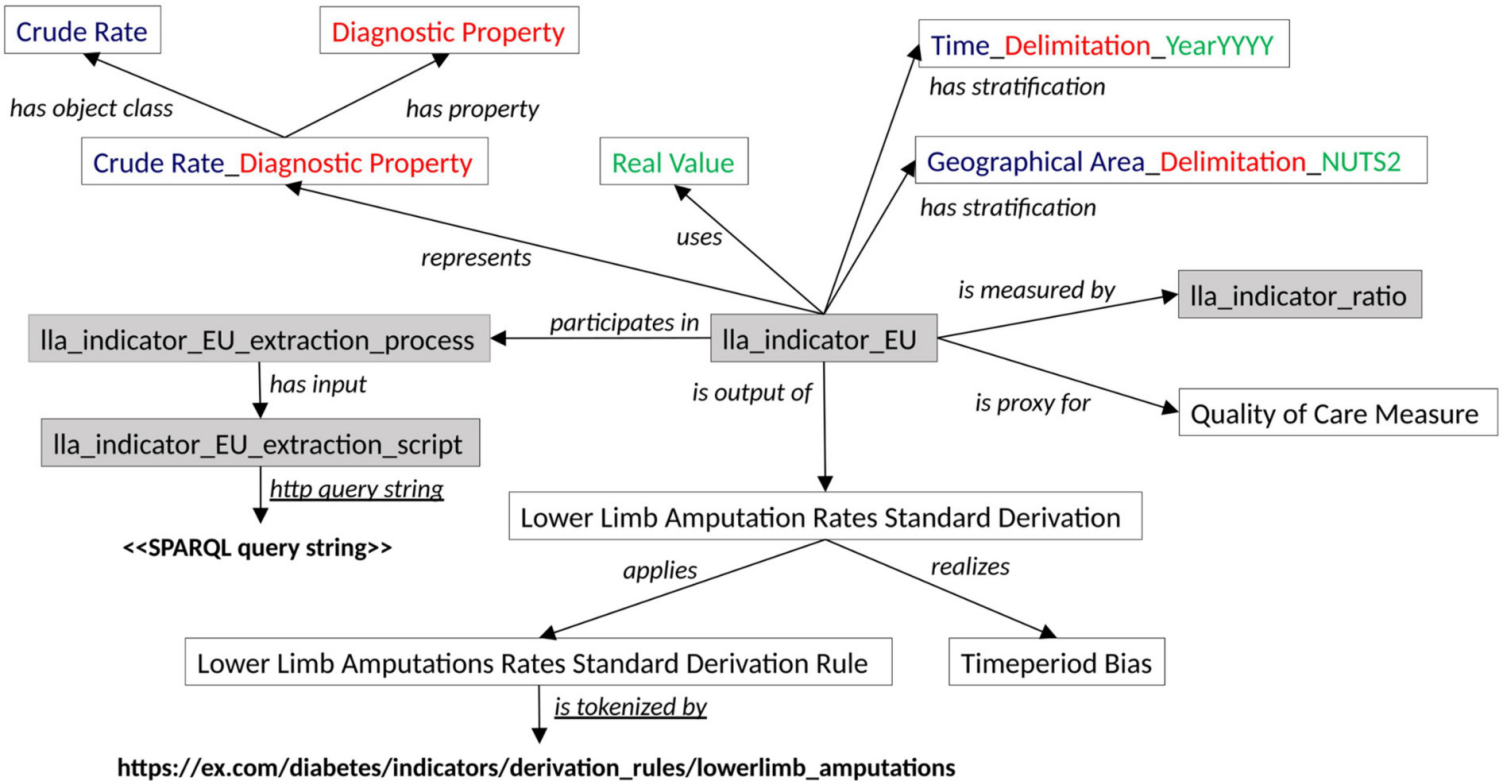
Network graph showing some of the SOLICIT relations involved in an indicator individual.

**Figure 2:**
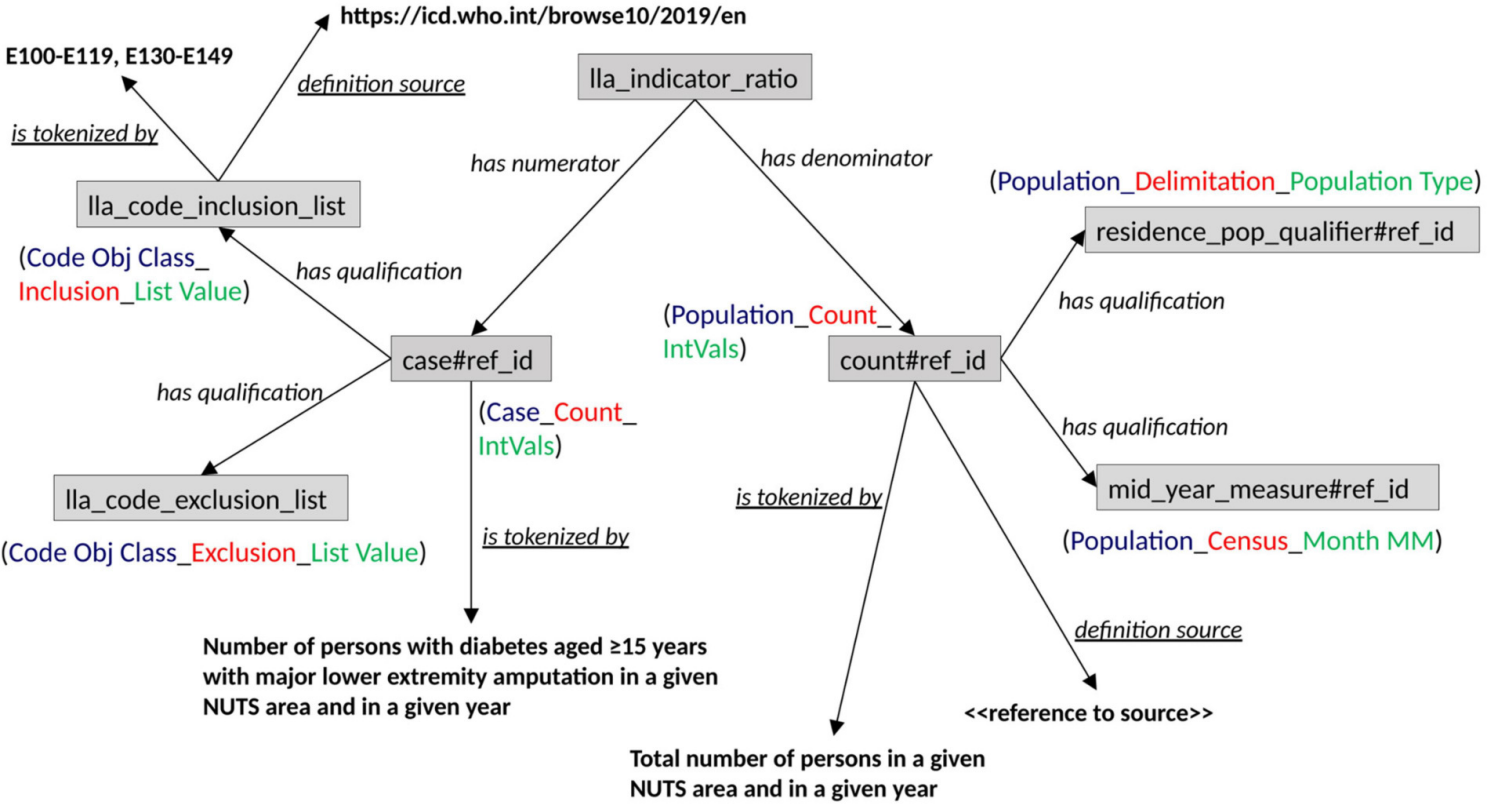
Network graph showing further SOLICIT relations involved in an indicator individual.

**Figure 3:**
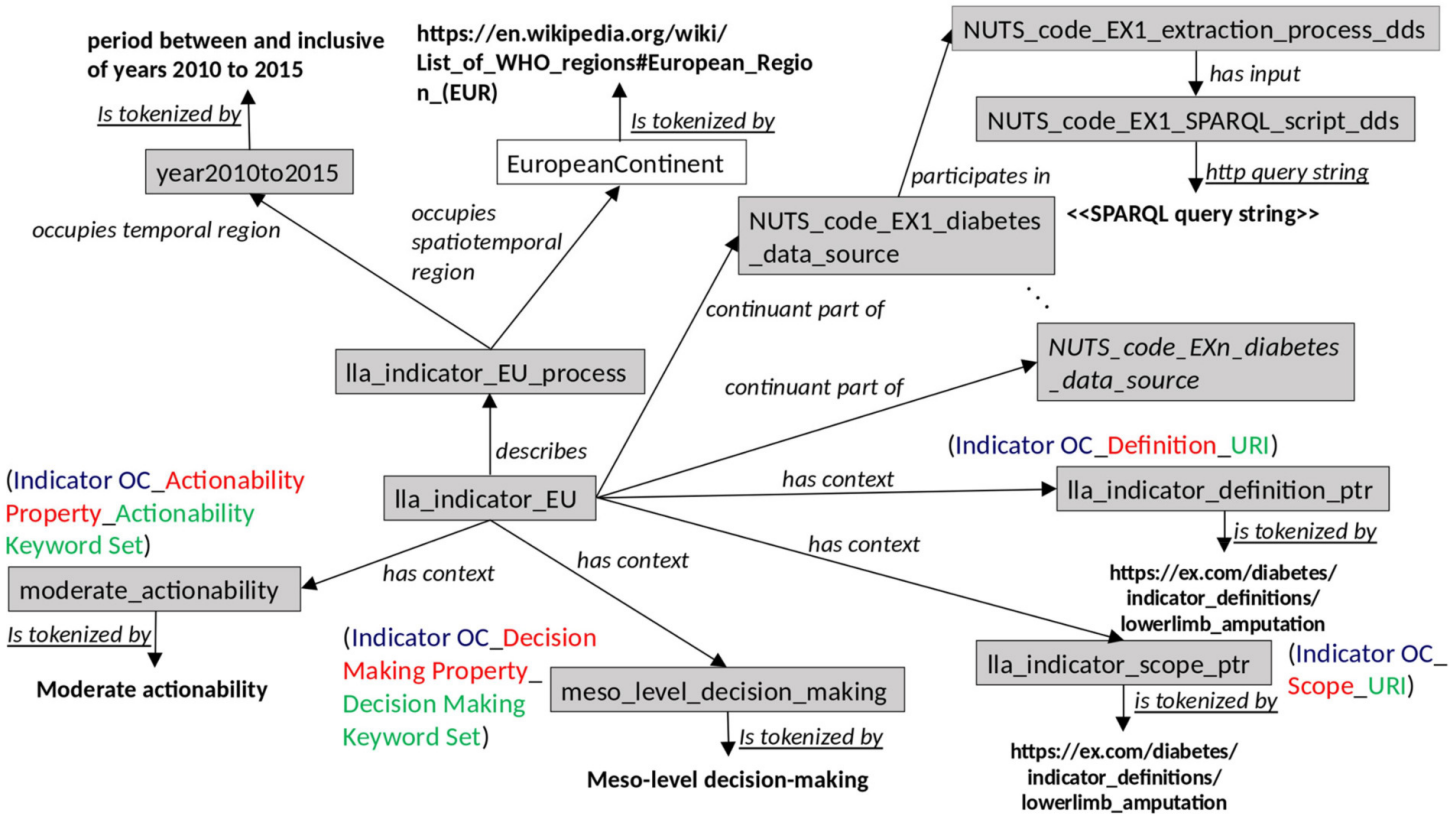
Network graph showing the remainder of the SOLICIT relations involved in an indicator individual.

Figures 1–3 illustrate how the contextual information for the lower limb amputation indicator can be built up within the context of the indicators selected by the CHIEF-diabetes design working group. The shaded boxes in the figure depict OWL individuals (or instances of OWL classes); unshaded boxes depict OWL classes; bold text indicates values; italicised text denotes OWL object or data properties (semantic relations); and underlined italicised text denotes OWL annotation properties. Compound text made up of different colours denotes the constituent parts of a metadata element in terms of the ISO/IEC 11179 entities: object class, property, and value domain. The semantic relations recognised by a SOLICIT indicator class are summarised in Table 2.

**Table 2:** Overview of the semantic relations recognised by a SOLICIT indicator class.

Semantic relation	Meaning	Inherited from a parent class?
Represents	ISO/IEC 11179 relation for describing a DEC	Yes
Uses	ISO/IEC relation for describing an ISO/IEC 11179 value domain	Yes
Is about	General description relation	Yes
Describes	An unbounded relation that SOLICIT uses to describe the indicator process (and via this, the temporal and spatiotemporal regions as foreseen by CCO).	Yes
Is measured by	Specifies a range of a CCO Measurement Information Content Entity class. It can be qualified further, dependent on the specific class (e.g. a ratio measurement can be further decomposed into numerator and denominator	No
Is proxy for	Describes what the indicator is a measure of	No
Is output of	Used to describe the standard method for deriving the indicator	No
Participates in	Used to extract the raw data file associated with the indicator (SOLICIT describes the metadata, allowing data users to understand the raw data)	No
Continuant part of	Allows data users to drill down to details regarding the data sets of the underlying data providers (if data providers have established a SPARQL endpoint).	No
Has qualification	Used when necessary to provide further qualification on a context element	No
Has context	A sub-relation of “has qualification”; it is a generic relation for any other contextual information not included in the other relations described above. The type of contextual information is dereferenced from the tripartite ISO/IEC 11179 metadata element composition	No
Has stratification	A sub-relation of “has qualification” that directly specifies a metadata element as a stratification variable of the indicator	No

The relations “represents” and “uses” (shown in Figure 1) are inherited from the indicator's parent OWL class and comply with the ISO/IEC 11179 relations describing respectively the DEC and the value domain of a data element. The object class is represented in blue text (“Crude Rate”) and the property is represented in red text (“Diagnostic Property”). The DEC therefore encapsulates the fact that the lower limb amputation indicator (“lla_indicator_EU”) is a diagnostic measure of a crude rate statistic (i.e. a raw ratio of numbers). The value domain is depicted in green text (“Real Value”) and denotes in what terms the DEC is measured. The two stratification variables of the indicator (time period and geographical area, cf. Figure 1) are described in terms of an ISO/IEC 11179 data element, and the coloured text again differentiates the composite nature of object class, property, and value domain. Other relations show that (i) the indicator is a ratio (with further information provided by the relations of the ratio class); (ii) it is a proxy for measuring quality of care; and (iii) it is the output of a derivation that applies the rule pointed to by the URL and has a potential time period bias (given a restrictive time period window on the cases being measured). The means of extracting the data from the raw indicator data file can be specified by means of a link to a suitable application or script, illustrated in Figure 1 by a reference to a dummy SPARQL script.

The “is measured by” relation (Figure 1) is used to specify how the indicator is measured. The entity lla_indicator_ratio has type ratio, and the numerator and denominator can be ascertained automatically by dereferencing the associated relations (cf., Figure 2), as well as any number of qualifications that may be attached to them (such as diagnostic codes and time of population census).

The relation “describes” (Figure 3) can be used to characterise the indicator process and reference other details such as the time period and the geographical coverage. The constituent data sources and how they can be accessed individually can also easily be specified via the generic relation “continuant part of”. Other contextual information can be added via the “has context” relations, where the related context is understandable from the ISO/IEC 11179 tripartite metadata elements and the specific values given via the CCO “is tokenized by” annotation relation.

There is, in effect, no limit on the contextual elements that can be added. All these elements can be dereferenced by data users on a need-to-know basis to provide them with the necessary information to take informed decisions on the usage of the indicator for a given purpose. Moreover, since all the elements are in machine-readable form and can point to standard terminology sources, the indicator context could be read by AI applications to make automatic inferences on the basis of the information provided.

## 4 Discussion

A concept of a generic metadata framework approach has been presented for realising a sustainable NCD indicator framework within CHIEF for a meaningful comparison of indicators across regional and national boundaries. The proposal contains novel ideas that have not been implemented before and have the potential for overcoming the limitations of previous approaches.

Regarding the first objective of providing a suitable generic metadata model, such a model should describe metadata in a form that can easily reuse terms, extend them, and map them to other entities, descriptions, and definitions in a scalable way. It should be suitable for a distributed platform of data sources (i.e. supporting data federation) and allow searches across federated data sources both within a disease domain and across disease domains. It should also provide a mechanism for semantic linkage to external classification schemes, including data dictionaries, thesauri, and ontologies. The proposed model should be easily maintainable and lead to reproducible results within the local context, particularly to allow regular repeated comparisons. Considering the general lack of availability of suitable frameworks, it was agreed to use the novel and recently proposed framework, SOLICIT, which supports most of these requirements.

**Figure 4:**
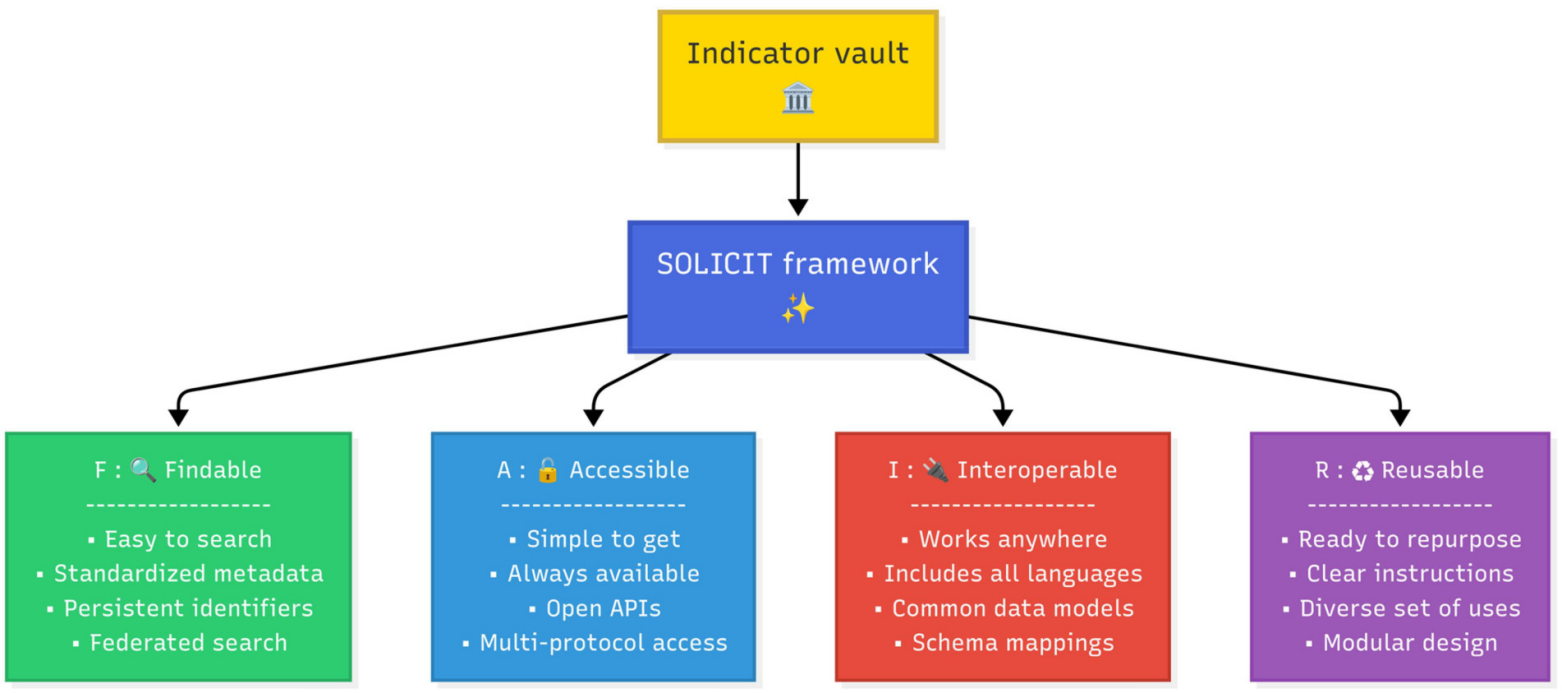
FAIR foundations of an effective indicator vault. Emoji icons from Unicode (https://home.unicode.org/): Classical Building, Sparkles, Magnifying Glass, Unlocked Lock, Electric Plug and Recycling Symbol, licensed under UNICODE LICENSE V3.

Regarding the second objective of conceptual model description, we presented a selection of necessary elements for a framework that would enable semantic linkage for all the descriptive components, with which the interoperability with other metadata models using FAIR data principles would be achieved. Such a framework would provide FAIR foundations for an effective indicator vault as depicted in Figure 4.

Such a model provides many advantages, including:

1. structuring and linking of contextual metadata according to a standard model;
2. description of disease domain specificities to any degree of granularity;
3. provision of a taxonomy of metadata elements that can be extended at the domain level to incorporate the associated specificities and map to existing definitions at a federated level;
4. annotation of general datasets with reference to the ontology metadata elements, which themselves can be linked to standard data dictionaries, thesauri, and ontologies;
5. conformance to an international standard (ISO/IEC 11179 metadata registry standard), providing a general description framework for data; and
6. facilitating interoperability between ontologies using a mid-level ontology layer, in the context of a distributed indicator framework, allowing for the reuse of a foundational set of semantic relations without the need of having to reinvent them each time.

In addition, it provides granular information to data users and AI-based applications that may not otherwise be in a position to know how the raw indicator values can be used or compared for a given purpose. The generic nature of the model allows its application to other disease domains beyond that of diabetes. The widespread implementation of the model would address the needs of a cross-section of different stakeholders through a full contextualisation of indicators, enabling downstream processes to understand the nature and limitations of the indicators and, more importantly, the suitability of comparing them in a meaningful sense. Current implementations reveal substantial gaps in this regard, as exemplified by international health portals like the WHO's Global Health Observatory (35). While methodologically precise, their reliance on standardised definitions for country-level data submission could conceal unstandardised local implementations and embedded contextual biases. This produces indicators documented as comparable yet suboptimal for end users—representing reactive, *post hoc* harmonisation that forces users to decipher methodological notes to understand comparison limits. Advanced features to support comparability have been surprisingly lacking in practically every existing indicator platform, where data users are obliged to accept that indicators are directly comparable regardless of the heterogeneity of the underlying assumptions, biases, and other limitations of different data providers. Effective comparability should be engineered for contextual insight as a foundational property of the system through coordinated governance and technology from the outset. An effective governance foundation should engage stakeholders in explicit definition negotiation, prioritise quality over convenience, and establish transparent, auditable decision trails. Simultaneously, technical execution should provide consistent, machine-actionable data representation, shifting practice from reactive reconciliation to proactive conformity with predefined, rigorous standards. Together, these components can create a self-reinforcing ecosystem where governance generates precise requirements, technical standards transform them into FAIR interoperable assets, and these assets continuously refine governance processes.

Beyond these immediate advantages, the model has some additional and far-reaching benefits that are elaborated in the following section.

### 4.1 Other benefits of the model

The mechanisms provided by SOLICIT also allow a versatile means for describing data quality and integration of standard data quality frameworks. A quality label can be attached to any data component or data process of a SOLICIT indicator (33), allowing retrieval of the whole chain of quality labels across the various data processing stages involved in the derivation of an indicator.

Depending on the quality framework employed, this history chain could provide an overall quality metric on the indicator, depending on the purpose for which the indicator is used. The overall quality metric could, in fact, be automatically derived from an appropriate set of axioms defined using DL. Greater transparency is thereby provided to any overall single data quality label since the quality provenance throughout all the underlying processing stages is available.

A further enhancement to the framework will be the mapping of the indicator context instances to an overall indicator quality metric that would give data users an immediate understanding of the comparability of similar indicators from different data sources.

The mechanisms provided by SOLICIT are also able to add appropriate functionality to common data models (CDMs) such as the Observational and Medical Outcomes Partnerships (OMOP) CDM. The OMOP CDM is designed to standardise the structure, format, and terminologies of datasets to facilitate systematic analyses across a federated network (36) of heterogeneous data.

Whereas CDMs are a key instrument for allowing interoperable data exchange, they do not capture the context or history of the datasets and have several disadvantages, including information loss, semantic inconsistency, and maintenance overheads (37). Moreover, even though they ensure data are in an interoperable form for a given analytical study, the study may be compromised as a result of unwarranted assumptions about the data or applicability. SOLICIT can provide such information.

This provides CHIEF with the potential to operate in a way that is complementary to the EHDS, where electronic health records (EHR) are increasingly aligned with the associated standards and are gaining importance as a primary data source for epidemiological analyses (38).

To improve interoperability of EHR and reduce maintenance costs, information systems in healthcare are moving away from single-model methodologies towards dual-model methodologies (39). Single-model methodologies refer to those that tightly couple domain concepts within the data model (generally using object-oriented or entity-relationship models). In contrast, dual-model methodologies structure information at two different levels; the first level consists of a non-volatile reference object model on which the software is developed, and the second level contains domain-level definitions in the form of archetypes (40).

The archetypes may change without necessitating rebuilds of the information system software since the latter depends only on more stable, generic concepts. The query interfaces are, however, radically different for information systems designed from these two types of methodologies and therefore pose challenges for data interoperability.

By mapping the data elements of both types of system to SOLICIT's Context Specifying Data Element class, the mapping mechanisms provided by SOLICIT could, in principle, merge the data for analytic purposes and extract the metadata without loss of meaning (via the contextual description classes). Using the same mapping mechanism, SOLICIT could, in fact, provide the means of adding contextual information to any archetype and thereby extend the utility of the concept and the comparability of archetypes provided by different data providers. Indeed, SOLICIT has the means to integrate heterogeneous data from any domain and facilitate the fusion of data sets for any purpose that can be justified from the contextual information and limitations of the data described within the framework.

Meaningful participatory design in such a complex environment of NCD indicators will be only possible at the European level with “tools to think with” providing a common language for all involved stakeholders. Only with their presence can there be an inclusive debate about what works when and why (41).

Therefore, the building of an effective model represents only a first step in the process towards the second one. Finally, usability in broad collaboration will be enhanced by the creation of visual tools such as indicator designers and rich representation platforms for a diverse list of end users of indicator information on the local, national, and European levels. The pursuit of comprehensive metadata governance, exemplified by the EHDS, presents a considerable challenge: the consolidation of diverse, legacy data formats into a coherent and interoperable framework. While an abundance of robust metadata is undeniably essential, a focused, indicator-driven metadata strategy is paramount to ensure documentation efforts are used to facilitate the derivation and application of meaningful key indicators, rather than diverting resources from their effective delivery.

The fundamental issue is prioritisation data within a sea of heterogeneous sources to avoid excessive documentation. The solution also requires optimisation at the point of entry. This should be achieved by aligning indicator developers with primary data collectors through shared governance forms, designing optimised standardised indicators directly into their workflows from the very start of the indicator design phase. Their insight can be used to indicate the critical aspects of metadata in different domains, where they possess specific experience in the field. This approach ensures that the entire process—from data consolidation to documentation—is guided by the common goal of population health improvement, rather than bureaucratic necessities.

### 4.2 Final recommendations for implementing the model

Based upon the results of the present qualitative study, we identified a set of seven recommendations for implementation in three different areas: (a) contextualisation of common data elements and indicators; (b) a generic contextualisation framework; and (c) implementation on the EU level.

As far as indicator contextualisation is concerned, we recommended the following to foster metadata utilisation in NCD indicators:

- The meaningful comparison of indicators across different sources and organisations shall consider relevant contextual information to foster the correct epidemiological interpretation.
- A common schema for the description and semantic linkage of metadata shall be applied at the level of CDEs, as the building block for the definition of indicators.

Regarding the generic contextualisation framework, it is essential to ensure the following:

- A standard generic indicator contextualisation framework should underpin the development of indicators in specific disease domains to facilitate metadata reuse and linkage with indicators in other domains.
- Ontology approaches such as SOLICIT should be considered to provide the necessary coverage for the generic semantic contextualisation of NCD indicators.

Finally, implementation at the EU level should be favoured by pursuing:

- Pilot test of the model on federated statistical engines for the creation of indicators within and across different NCD domains.
- Development of European portals for the representation of NCD indicators.
- Implementation of a user-friendly interface as a front-end to semantic ontology approaches, e.g. SOLICIT, to enable data providers to contextualise data elements, avoiding the complexity of underlying ontologies.

The above recommendations can be used to foster the application of FAIR principles in EU health information systems for NCDs.

### 4.3 Limitations of the present study

This study presents relevant limitations that are worth addressing:

- The proposed approach is fairly complex, and the metadata model specified may be difficult to explain to different audiences. This also emerged as a major challenge during the creation of the conceptual model specified by the multidisciplinary panel of experts. The adoption of the model requires close and challenging cooperation between participating experts possessing diverse knowledge and different means of communication. While these limitations exist, they are expected to be manageable in implementation, since the model should operate through a user-friendly software solution that will present different user interfaces to each individual type of user based on role and required functionality.
- The results emerged from a discussion within a limited group of experts, which may make the responses to the research questions relatively subjective. However, we have continuously supported our discussion with objective references and regulations that are currently enforced at the EU level and require immediate action by targeted stakeholders. Experts are relevant leaders in the field of registries who can provide valuable help in the implementation of the stated concepts.
- The proposed concept is only in an early development phase. In the future, the functionalities of the framework will be demonstrated in a prototype, and the standard indicator contexts will continue to be developed. Additionally, consensus will be sought across different NCD domains, as well as the statistical toolkits for helping align indicators derived using different data sources.
- The adoption of any framework, including the present proposal, ultimately rests with each Member State unless enacted through binding legislation. This proposal is intended to stimulate debate and pave the way for a common consensus, establishing a foundational starting position.

## 5 Conclusions

In this qualitative study, we have presented the results of a concerted assessment between a multidisciplinary panel of experts, actively contributing to a European network of diabetes registries (1).

Currently, the collaboration between different disease registry networks is hampered by technical barriers to linking domain knowledge and associated concepts related to existing data models.

The proposed metadata model could provide the basis for shared knowledge management about NCD indicators and their origin within and between registry networks.

In this way, a common EU information system can be built on top of a flexible and disease domain-agnostic bridge from CHIEF to the EHDS, which can be directly scaled up to cover a spectrum of applications, from data discovery functions to the actionable use of indicators for an audience of multiple stakeholders, particularly people affected by chronic diseases.

This process will pave the way for the modernisation of current mostly static registry data dictionaries with many possible improvements of their functionality. Further research will be needed to test and validate the proposed mechanisms, using FAIR principles for the implementation of the EHDS.

## Data Availability

The original contributions presented in the study are included in the article; further inquiries can be directed to the corresponding author.
